# Supplementary material for: Evolution of SARS-CoV-2 Spikes shapes their binding affinities to animal ACE2 orthologs
Source: Microbiol Spectr. 2023 Nov 9;11(6):e02676-23. doi: 10.1128/spectrum.02676-23 (PMC10715038; doi:10.1128/spectrum.02676-23)
Supplement: Supplemental file 1 — Fig. S1 to S9 and Tables S1 to S5. [file spectrum.02676-23-s0001.pdf]

Evolution of SARS-CoV-2 Spikes shapes their binding affinities to animal ACE2 orthologs

Weitong Yao[a,b,c,1,2], Yujun Li[b,1,2], Danting Ma[b,g,1], Xudong Hou[a,b], Haimin Wang[i], Xiaojuan Tang[a,b], Dechun Cheng[b,h], He Zhang[a,b], Chengzhi Du[a,b], Hong Pan[a,b], Chao Li[b], Hua Lin[e], Mengsi Sun[b], Qiang Ding[f], Yingjie Wang[b,2], Jiali Gao[a,b,d,2], Guocai Zhong[a,b,i,j,k,2]

[a] School of Chemical Biology and Biotechnology, Peking University Shenzhen Graduate School, Shenzhen 518055, China
[b] Shenzhen Bay Laboratory, Shenzhen 518132, China
[c] Hubei JiangXia Laboratory, Wuhan, Hubei 430200, China (Current Affiliation)
[d] Department of Chemistry and Supercomputing Institute, University of Minnesota, MN 55455, USA
[e] Biomedical Research Center of South China, Fujian Normal University, Fuzhou 350117, China
[f] Center for Infectious Disease Research, School of Medicine, Tsinghua University, Beijing 100084, China
[g] Tianjin Medical University Chu Hsien-I Memorial Hospital, Tianjin 300134, China (Current Affiliation)
[h] Heilongjiang Academy of Medical Sciences, Harbin 150086, China
[i] Horae Gene Therapy Center, University of Massachusetts Chan Medical School, Worcester, MA 01605, USA. (Current Affiliation)
[j] RNA Therapeutics Institute, University of Massachusetts Chan Medical School, Worcester, MA 01605, USA. (Current Affiliation)
[k] Department of Biochemistry and Molecular Biotechnology, University of Massachusetts Chan Medical School, Worcester, MA 01605, USA. (Current Affiliation)
[1] Equal contribution
[2] Correspondence to guocai.zhong@umassmed.edu (G.Z.); liyujun@szu.edu.cn (Y.L.); yaowt@hbjxlab.com (W.Y.); wangyj@szbl.ac.cn (Y.W.); gao@jialigao.org (J. G.)

This PDF file includes:

Legends for Figures S1 to S9

Figs S1 to S9

Tables S1 to S5

SUPPLEMENTARY FIGURES AND LEGENDS: S1-S10

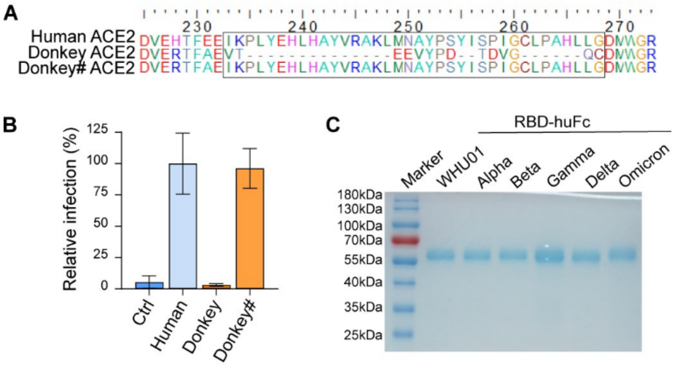

Fig S1. Fixed donkey ACE2 protein supports SARS-CoV-2 pseudovirus entry. (**A**) Amino-acid sequence alignment for human ACE2, donkey ACE2 and donkey ACE2#, from residue 225 to 273. The numbering is based on human ACE2 protein. Donkey ACE2# is a fixed donkey ACE2 (NCBI Reference Sequence ID: XM_014857647.1) that carries human ACE2 residues 233-268 as shown in the rectangle. (**B**) 293T cells expressing human, donkey, or donkey# ACE2 were infected with SARS-CoV-2 WHU01 Spike-pseudotyped reporter virus. Pseudovirus entry-mediated luciferase reporter expression was measured at 48 hours post infection and presented as percentage of infection relative to the infection signals observed with human ACE2. (**C**) SDS-PAGE images of purified human IgG1 Fc fusion proteins of the indicated SARS-CoV-2 variant RBDs (RBD-huFc) used in the S2 Fig experiments. Data shown in B are representative of two independent experiments with similar results, and data points represent mean ± s.d. of three biological replicates.

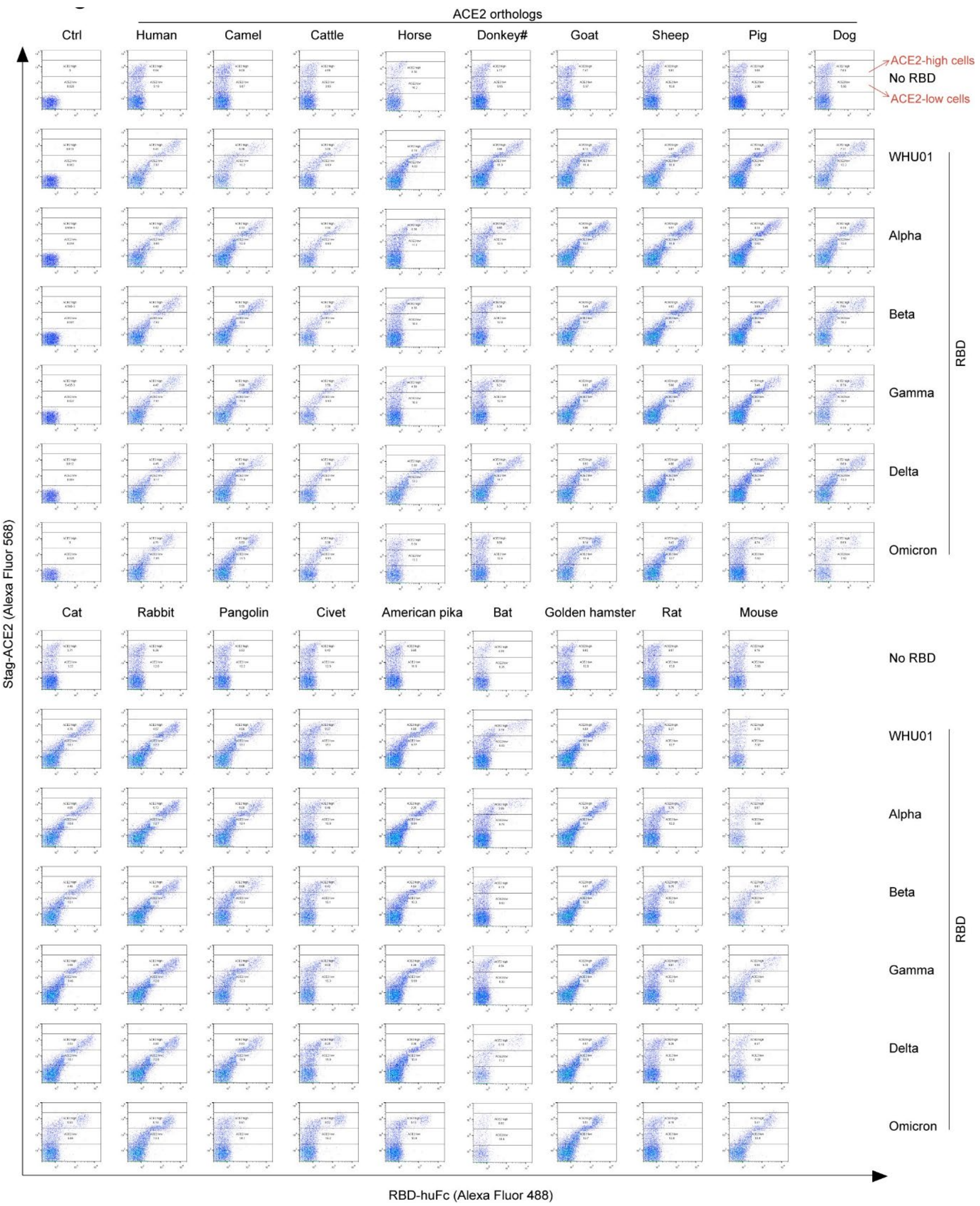

Fig S2. Flow cytometry detection of the interactions between the indicated RBD dimers and cell surface expressed animal ACE2 orthologs. 293T cells expressing one of the eighteen animal ACE2 orthologs were double stained for ACE2 protein expression as well as binding by the RBD-huFc proteins shown in S1C Fig. Cell were then analyzed using flow cytometry and dot plot raw data for all the samples are shown. ACE2 positive cells were gated into ACE2-high and ACE2-low populations and mean fluorescence intensity (MFI) of RBD binding signals for the two cell populations are separately analyzed in Figure 2. Data shown are representative of two independent experiments with similar results.

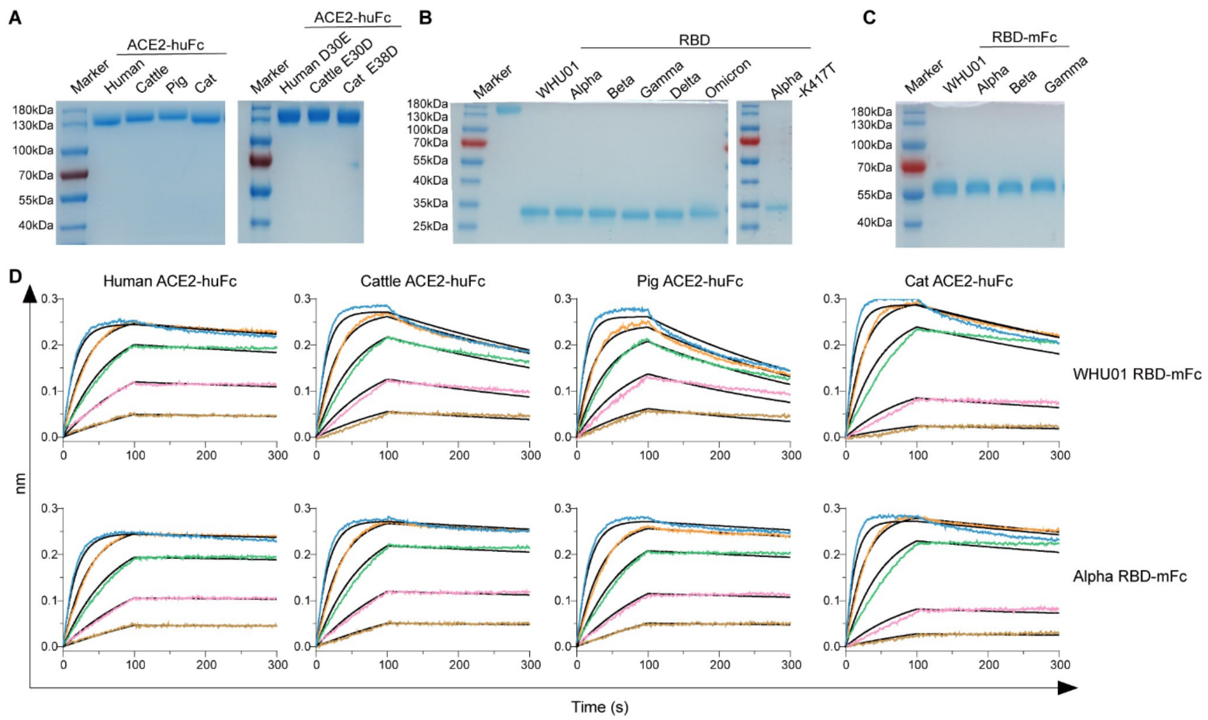

Fig S3. Alpha RBD has markedly enhanced affinity to cattle and pig ACE2 proteins. (**A-C**) SDS-PAGE images of purified human IgG1 Fc fusion proteins of the indicated animal ACE2 orthologs or their mutants (A), purified RBD monomer proteins (B), and purified mouse IgG2a Fc fusion proteins of the indicated SARS-CoV-2 variant RBDs (RBD-mFc) used in the following experiments or the experiments shown in Figure 3 and S3D. (**D**) Bio-layer interferometry (BLI) measurements of interaction kinetics were performed using one of the indicated animal ACE2 ectodomain dimers (ACE2-huFc) as immobilized ligand and one of the indicated RBD-mFc proteins at 100 nM, 50 nM, 25 nM, 12.5 nM, or 6.25 nM as analytes. The raw curves are shown in colors and the fitted curves obtained from a 1:1 Langmuir binding model (global fit) are presented in black. Data shown in D are representative of at least two independent experiments with similar results.

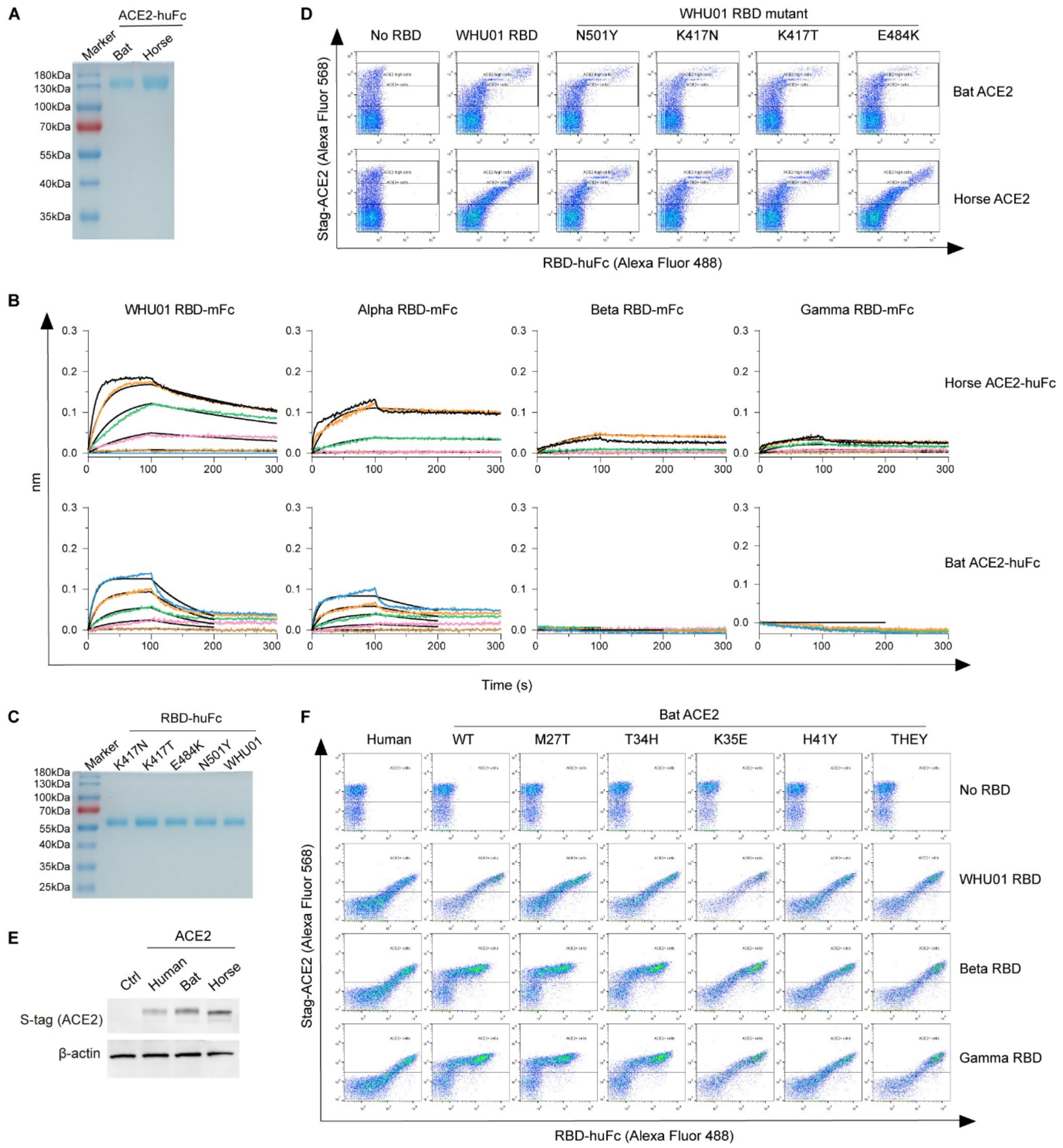

Fig S4. Beta and Gamma RBDs have lost affinity to *Rhinolophus sinicus* bat and horse ACE2 orthologs. (**A**) SDS-PAGE images of purified human IgG1 Fc fusion proteins of *Rhinolophus sinicus* bat and horse ACE2 ectodomains (ACE2-huFc) used in the following experiments. (**B**) BLI measurements were performed using one of the indicated ACE2-huFc proteins as immobilized ligand and one of the indicated RBD-mFc proteins at 100 nM, 50 nM, 25 nM, 12.5 nM, or 6.25 nM as analytes. The raw curves are shown in colors and the fitted curves obtained from a 1:1 Langmuir binding model (global fit) are presented in black. (**C**) SDS-PAGE images of purified human IgG1 Fc fusion proteins of the indicated SARS-CoV-2 WHU01 RBD mutants (RBD-huFc) used in panel D experiments. (**D**) Dot plot raw data for the flow cytometry data shown in Figure 4C. (**E**) 293T cells transfected with the indicated ACE2 genes (S-tagged) were collected at 24 hours post transfection. Expression levels of the ACE2 proteins were detected using Western Blot with an anti-S-tag antibody. (**F**) Dot plot raw

data for the flow cytometry data shown in Figure 4F. Data shown in B, D and F are representative of two independent experiments with similar results.

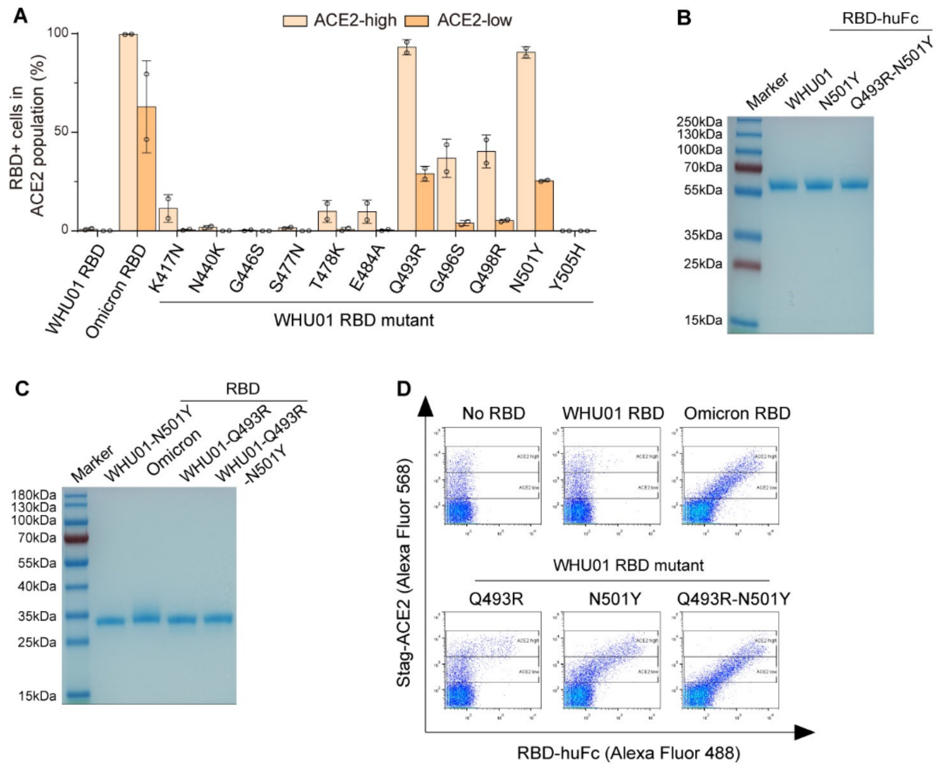

Fig S5. Omicron RBD efficiently binds to mouse ACE2 mainly through the RBD Q493R, G496S, Q498R, and N501Y mutations. (**A**) Mouse ACE2-high and ACE2-low cells in S7 Fig were separately analyzed for percentage of RBD positive cells in each analyzed cell population. (**B**) SDS-PAGE images of purified human IgG1 Fc fusion proteins of the indicated RBD variants (RBD-huFc) used in Figure 5E. (**C**) SDS-PAGE images of purified monomeric RBD proteins used in Figure 5F. (**D**) Dot plot raw data for the flow cytometry data shown in Figure 5G. Data shown in A, B and E are representative of two independent experiments with similar results.

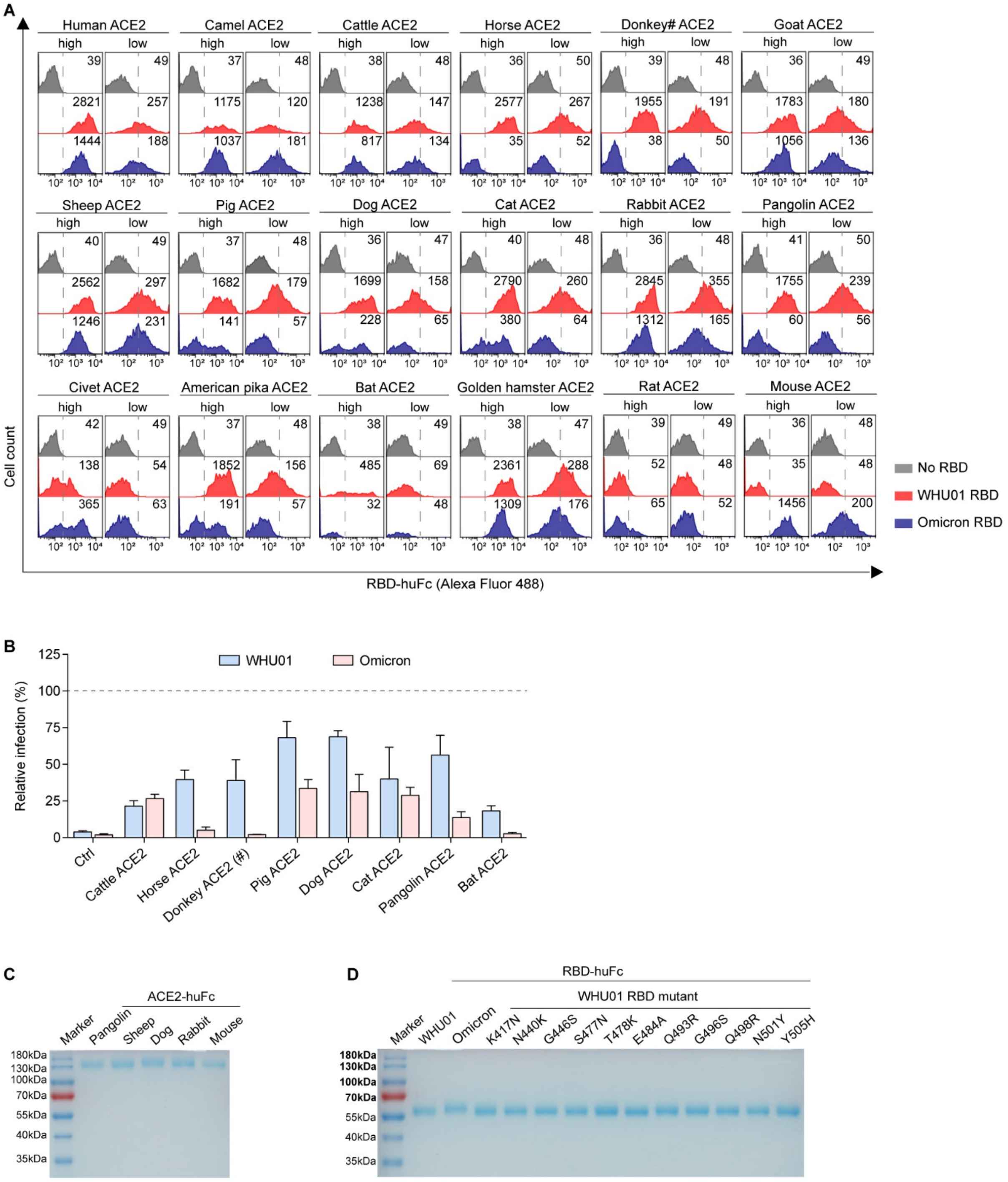

Fig S6. Omicron has lost affinity to eight tested ACE2 orthologs. (**A**) Flow cytometry histogram data obtained from the experiments shown in Figure 2 are shown for the indicated RBD variants (WHU01 and Omicron) binding to cell surface-expressed animal ACE2 orthologs. (**B)** 293T cells expressing the indicated ACE2 orthologs were infected with SARS-CoV-2 WHU01 or Omicron pseudovirus. ACE2-mediated pseudovirus entry was measured by a luciferase reporter expression at 48 hours post infection. Data points represent mean ± s.d. of three biological replicates. Infection signals of each pseudovirus supported by animal ACE2 were calculated as percentage of infection relative to signals of corresponding pseudovirus supported by human ACE2. **(C-D**) SDS-PAGE images of some purified ACE2-huFc proteins

used in Figure 7A and human IgG1 Fc fusion proteins of the indicated RBD variants (RBD-huFc) used in Figure 7B. Data shown are representative of two independent experiments with similar results.

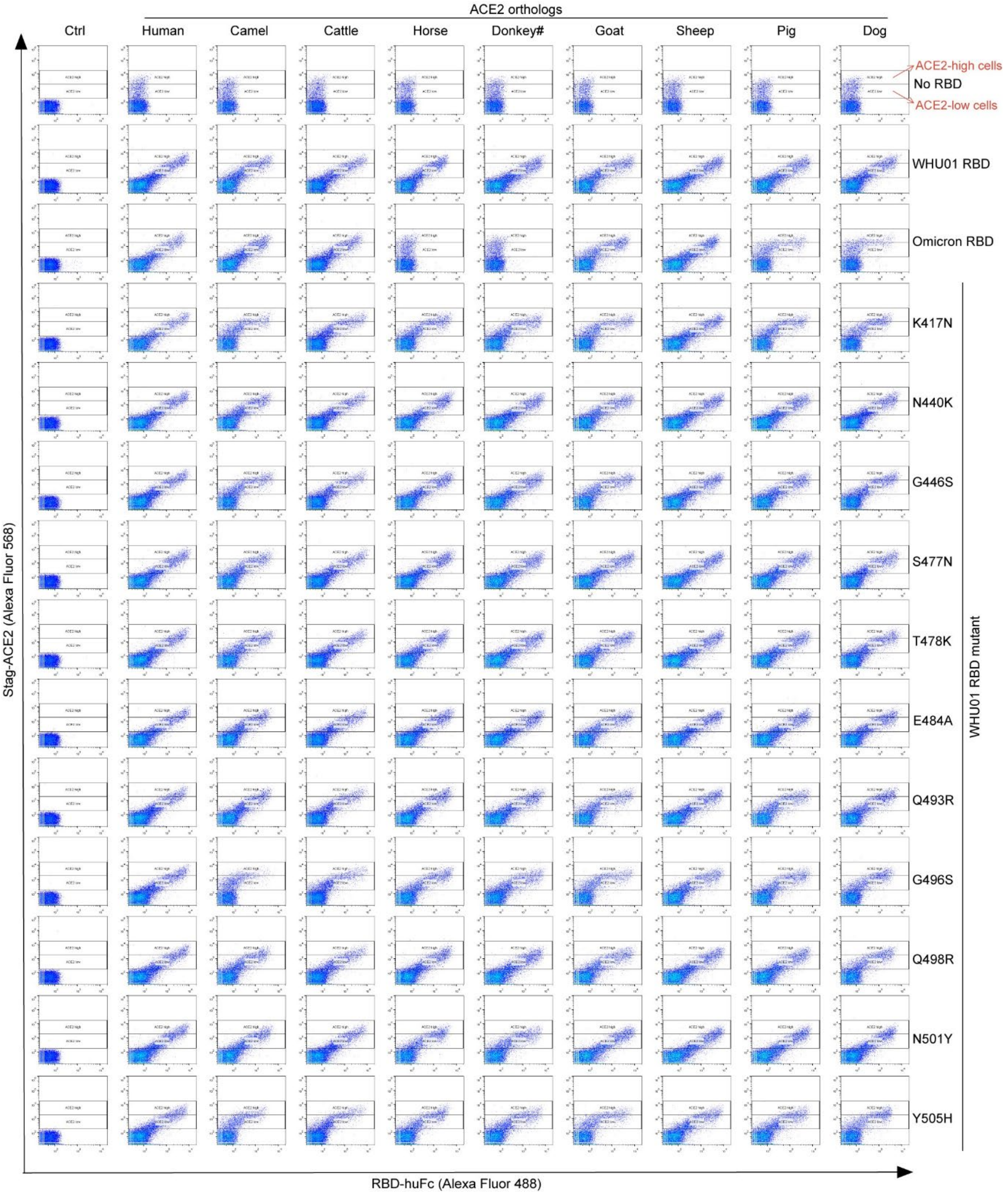

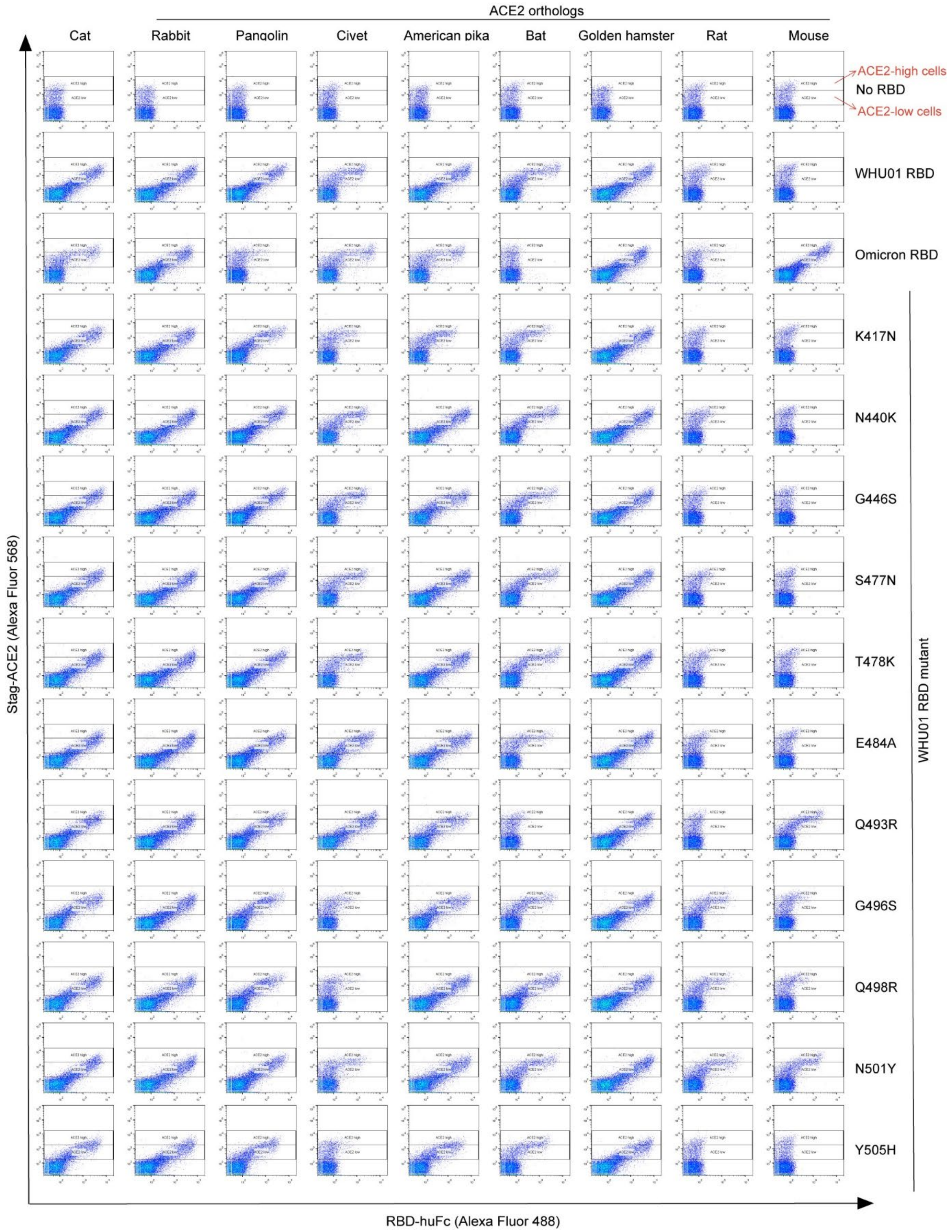

Fig S7. Dot plot raw data for the flow cytometry data shown in Figures 7B and S8. Flow cytometry detection of interactions between the indicated RBD dimers and cell surface expressed animal ACE2 orthologs. RBD binding signals in ACE2-high and ACE2-low populations were separately analyzed. Data shown in are representative of two independent experiments with similar results.

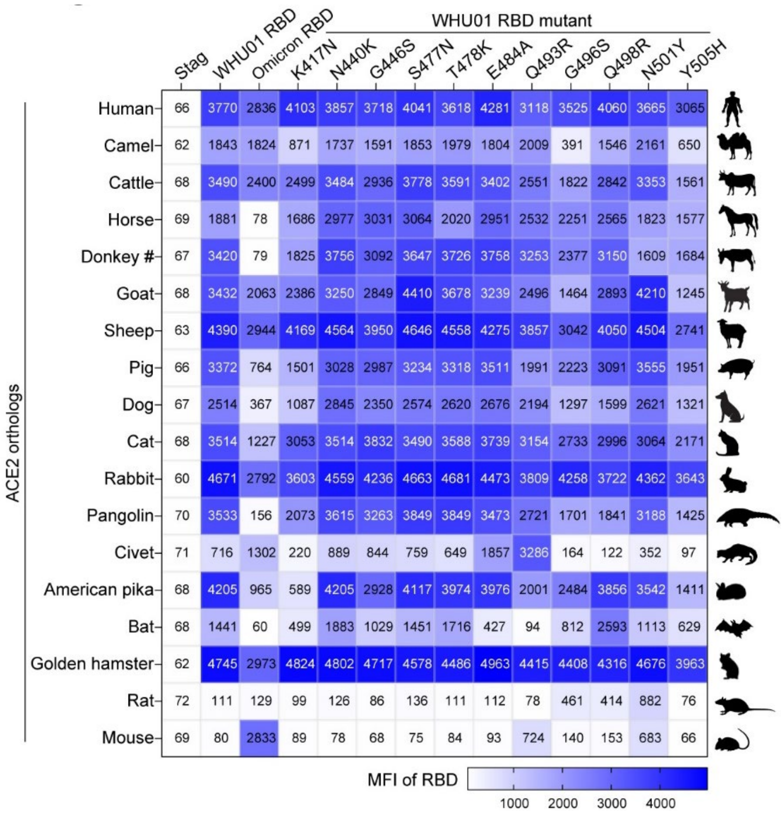

Fig S8. RBD binding signals in ACE2-high populations of the flow cytometry experiments shown in S7 Fig. MFI values for each RBD/ACE2 interaction are shown in color-coded heatmap grids. Data shown are representative of two independent experiments with similar results.

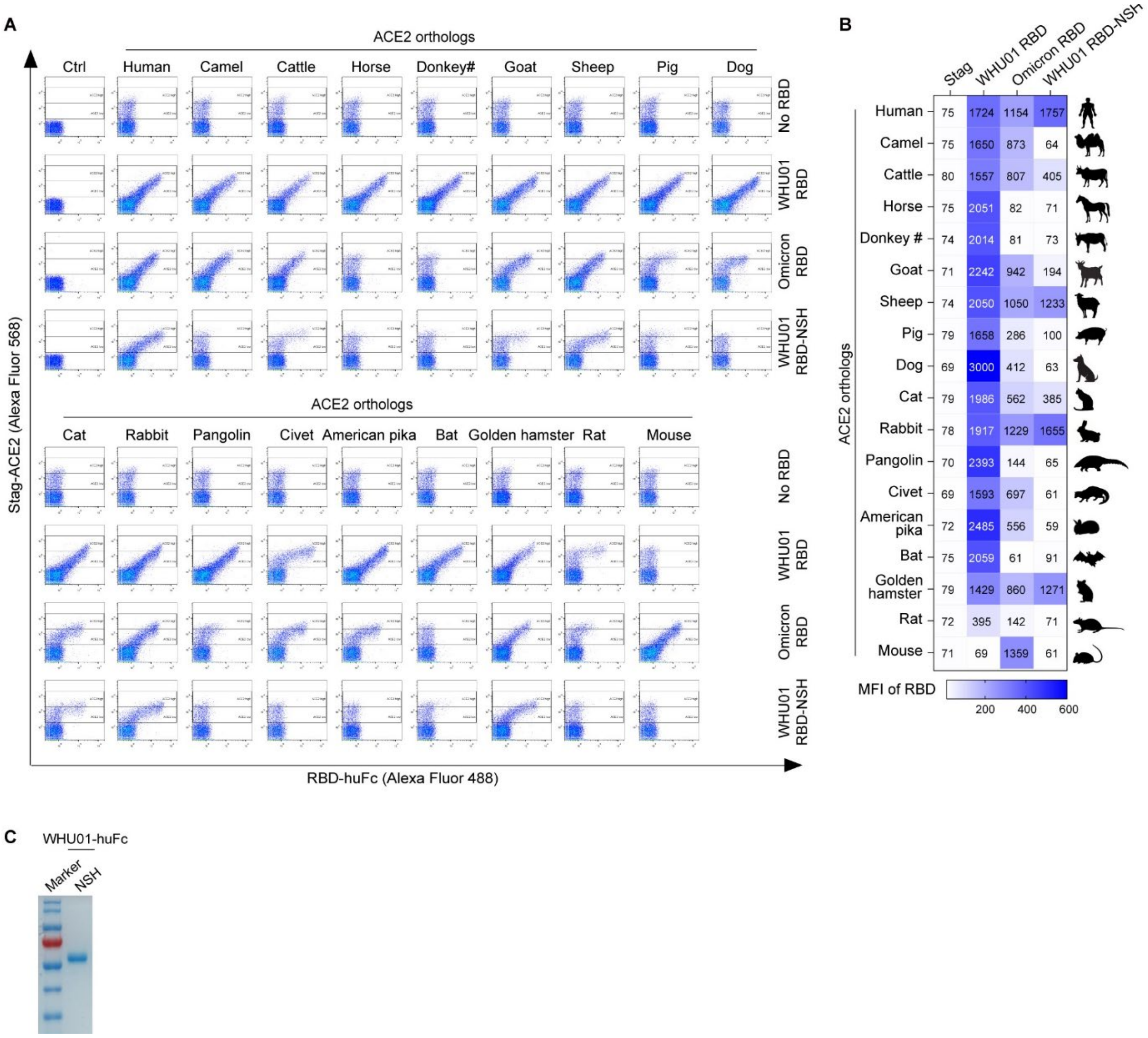

Fig S9. Flow cytometry detection of interactions between the indicated RBD dimers and cell surface expressed animal ACE2 orthologs. (**A**) Dot plot raw data for the flow cytometry data shown in Figure 7D and S9B. (**B**) RBD binding signals in ACE2-high populations of the flow cytometry experiments shown in A and Figure 7D. MFI values for each RBD/ACE2 interaction are shown in color-coded heatmap grids. (**C**) SDS-PAGE images of purified RBD-huFc proteins used in A. Data shown are representative of three independent experiments with similar results.

SUPPLEMENTARY TABLES: S1-S5

Table S1. Species and accession numbers of ACE2 orthologs

No.	Species name used in the text	Binomial name	NCBI Reference Sequence ID or Genebank ID
1	Human	Homo sapiens	NM_021804.3
2	Camel	Camelus bactrianus	XM_010968001.1
3	Cattle	Bos Taurus	NM_001024502.4
4	Horse	Equus caballus	XM_001490191.5
5	Donkey	Equus asinus	XM_014857647.1
6	Goat	Capra hircus	NM_001290107.1
7	Sheep	Ovis aries	XM_012106267.3
8	Pig (Domestic)	Sus scrofa domesticus	XM_021079374.1
9	Dog	Canis lupus familiaris	NM_001165260.1
10	Cat (Domestic)	Felis catus	NM_001039456.1
11	Rabbit	Oryctolagus cuniculus	XM_002719845.3
12	Pangolin	Manis javanica	XM_017650257.1
13	Civet	Paguma larvata	GQ262789.1
14	American pika	Ochotona princeps	XM_004597492.3
15	Bat	Rhinolophus sinicus	KC881004.1
16	Golden hamster	Mesocricetus auratus	XM_005074209.2
17	Rat	Rattus norvegicus	NM_001012006.1
18	Mouse	Mus musculus	NM_027286.4

Table S2. SPR data for the interaction of ACE2 with SARS-CoV-2 RBDs

Loading Sample (ACE2-hFc)	Analytes (RBD-monomer)	Rmax (RU)	k_a ($\times 10^5$ M-1 s-1)	k_d ($\times 10^{-3}$ s-1)	K_D (nM)
Human	WHU01	41.3	0.92	13.7	129
Human	Alpha	46.6	1.17	3	25.7
Cattle	WHU01	66.8	0.94	66	700
Cattle	Alpha	61.4	1.26	6	47.6
Pig	WHU01	64.2	0.87	100	1140
Pig	Alpha	59.4	1.14	9.6	84.4
Cat	WHU01	74.4	0.7	55.1	792
Cat	Alpha	69.1	0.96	28.7	299
Human D30E	WHU01	332.3	1.02	8.9	87.7
Human D30E	Alpha	304.9	1.5	1	6.6
Cattle E30D	WHU01	165.2	0.36	46.7	1290
Cattle E30D	Alpha	159.6	0.67	14.8	223
Cat E38D	WHU01	168	0.57	36.3	635
Cat E38D	Alpha	164.4	0.65	6.8	105
Human	WHU01-K417T-N501Y	151.3	0.625	3.54	56.6
Human D30E	WHU01-K417T-N501Y	142.2	0.735	2.02	27.5

Table S3. SPR data for the interaction of Bat and Horse ACE2 with SARS-CoV-2 RBDs

Loading Sample (ACE2-hFc)	Analytes (RBD-monomer)	Rmax (RU)	ka ($\times 10^5$ M-1 s-1)	kd ($\times 10^{-3}$ s-1)	KD (nM)
Bat	WHU01	142.2	0.3	34.3	1140
Bat	Alpha	306.5	N.D.	N.D.	N.D.
Bat	Beta	307.6	N.D.	N.D.	N.D.
Bat	Gamma	305.5	N.D.	N.D.	N.D.
Horse	WHU01	198.9	1.41	9.5	67.4
Horse	Alpha	212.2	N.D.	N.D.	N.D.
Horse	Beta	332.3	N.D.	N.D.	N.D.
Horse	Gamma	304.9	N.D.	N.D.	N.D.

Table S4. SPR data for the interaction of Mouse ACE2 with SARS-CoV-2 RBDs

Loading Sample (ACE2-hFc)	Analytes (RBD-monomer)	Rmax (RU)	ka ($\times 10^5$ M-1 s-1)	kd ($\times 10^{-3}$ s-1)	KD (nM)
Mouse	WHU01	2.5	N.D.	N.D.	N.D.
Mouse	Alpha	7.9	N.D.	N.D.	N.D.
Mouse	Beta	113.1	0.71	234	3280
Mouse	Gamma	97	0.71	145	2060
Mouse	Delta	9	N.D.	N.D.	N.D.
Mouse	Omicron	118.1	2.22	52.6	237
Mouse	WHU01-Q493R	42.7	N.D.	N.D.	N.D.
Mouse	WHU01-N501Y	59.4	N.D.	N.D.	N.D.
Mouse	WHU01-Q493R-N501Y	84.1	10300	70300	68

Table S5 . SPR data for the interactions between animal ACE2 orthologs and two RBD variants

Loading Sample (ACE2-hFc)	Analytes (RBD-monomer)	Rmax (RU)	ka ($\times 10^5$ M-1 s-1)	kd ($\times 10^{-3}$ s-1)	KD (nM)
Human	WHU01	141.6	1	13.4	133
Human	Omicron	143.9	0.8	8.3	104
Horse	WHU01	118	0.5	4.7	89.8
Horse	Omicron	0.8	N.D.	N.D.	N.D.
Pig	WHU01	153.2	0.4	39.5	1000
Pig	Omicron	205.6	0.2	411	18200
Dog	WHU01	140.3	0.6	17.6	275
Dog	Omicron	7753.8	0	227	419000
Cat	WHU01	172.3	0.7	37.5	554
Cat	Omicron	289.1	0.3	337	13400
Pangolin	WHU01	107	447	3000	67.1
Pangolin	Omicron	41.6	0	2.3	938
Bat	WHU01	155.5	0.2	37.7	1920
Bat	Omicron	0	N.D.	N.D.	N.D.
Human	Omicron	115.4	2.72	8.42	31.0
Human	Omicron-R493Q	110.0	0.374	0.79	21.1
Human	Omicron-S496G	119.1	3.15	3.5	11.1
Human	Omicron-R493Q/S496G	119.5	1.03	0.428	4.15
